# Di-μ-methanolato-bis[(2-*tert*-butyl-6-methylphenolato-κ*O*)methyl­titanium(IV)]

**DOI:** 10.1107/S1600536813025634

**Published:** 2013-09-21

**Authors:** Alastair J. Nielson, Chaohong Shen, Joyce M. Waters

**Affiliations:** a Chemistry, Institute of Natural and Mathematical Sciences, Massey University at Albany, PO Box 102904 North Shore Mail Centre, Auckland, New Zealand; b Formerly Chemistry, Institute of Fundamental Sciences, Massey University at Albany, PO Box 102904 North Shore Mail Centre, Auckland, New Zealand

## Abstract

The molecule of the title compound, [Ti_2_(CH_3_)_2_(CH_3_O)_2_(C_11_H_15_O)_4_] or {[Ti(Me)(μ-OCH_3_)(OC_6_H_3_CMe_3_-2-Me-6)]_2_}, has a centrosymmetric, dimeric structure with a distorted square pyramidal array about each titanium atom. The methoxide ligands form an asymmetric bridge between the two Ti^IV^ atoms [Ti—O bond lengths of 1.9794 (12) and 2.0603 (12) Å] with the two phenolato ligands occupying the remaining basal sites [Ti—O 1.8218 (11) and 1.8135 (11) Å]. The Ti—O—C phenolato bond angles are similar at 161.24 (10) and 160.66 (11)°. The methyl ligand attached to the metal atom has a Ti—C bond length of 2.0878 (17) Å.

## Related literature

For other alkoxy-bridged dialkyl or diphenyl bis-phenolate dititanium complexes, see: Janas *et al.* (2004 ▶, 2005 ▶); Zhang (2007*a* ▶,*b* ▶); Kobylka *et al.* (2007 ▶). For other alk­oxy-bridged bis-phenolato dititanium complexes, see: Ejfler *et al.* (2004 ▶). For insertion of oxygen into a terminal Ti—Me bond to give a μ-meth­oxy ligand, see: Zhang (2007*a* ▶. For general phenolato and alkyl­ato complexes, see; Bradley *et al.* (1978 ▶). For bis-phenolato complexes of titanium containing 2-(1,1-di­methyl­eth­yl) and 6-methyl subsituents, see: Nielson *et al.* (2005 ▶); Santora *et al.* (1999 ▶). For some crystal structures of titanium complexes containing terminal and bridging phenolato ligands, see: Gowda *et al.* (2009 ▶); Nielson *et al.* (2006 ▶); Svetich & Voge (1972 ▶).
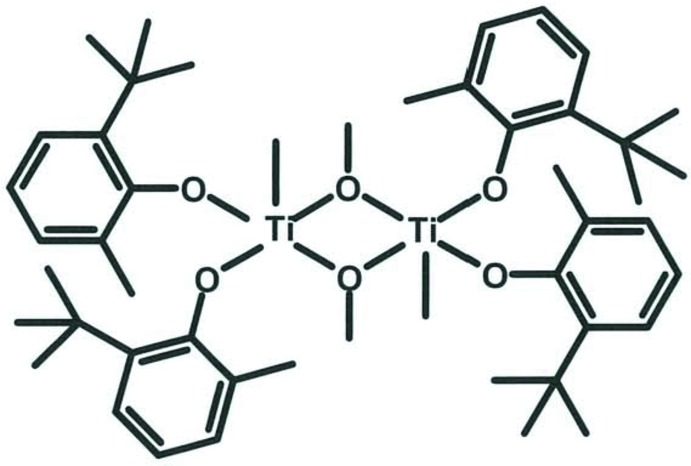


## Experimental

### 

#### Crystal data

[Ti_2_(CH_3_)_2_(CH_3_O)_2_(C_11_H_15_O)_4_]
*M* *_r_* = 840.80
Monoclinic, 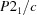

*a* = 15.127 (3) Å
*b* = 11.067 (2) Å
*c* = 15.821 (3) Å
β = 115.71 (3)°
*V* = 2386.4 (8) Å^3^
*Z* = 2
Mo *K*α radiation
μ = 0.38 mm^−1^
*T* = 150 K
0.48 × 0.20 × 0.18 mm

#### Data collection

Siemens SMART diffractometer
Absorption correction: multi-scan (Blessing, 1995 ▶) *T*_min_ = 0.834, *T*_max_ = 0.953
13277 measured reflections
4844 independent reflections
4113 reflections with *I* > 2σ(*I*)
*R*_int_ = 0.022

#### Refinement

*R*[*F* ^2^ > 2σ(*F* ^2^)] = 0.034
*wR*(*F* ^2^) = 0.089
*S* = 1.04
4844 reflections
263 parameters
H-atom parameters constrained
Δρ_max_ = 0.30 e Å^−3^
Δρ_min_ = −0.30 e Å^−3^

### 

Data collection: *SMART* (Siemens, 1995 ▶); cell refinement: *SAINT* (Siemens, 1995 ▶); data reduction: *SAINT*; program(s) used to solve structure: *SHELXS93* (Sheldrick, 2008 ▶); program(s) used to refine structure: *SHELXL97* (Sheldrick, 2008 ▶); molecular graphics: *ORTEP-3 for Windows* (Farrugia, 2012 ▶); software used to prepare material for publication: *SHELXL97*.

## Supplementary Material

Crystal structure: contains datablock(s) I, global. DOI: 10.1107/S1600536813025634/gg2122sup1.cif

Structure factors: contains datablock(s) I. DOI: 10.1107/S1600536813025634/gg2122Isup2.hkl

Additional supplementary materials:  crystallographic information; 3D view; checkCIF report

## Figures and Tables

**Fig. 1.**
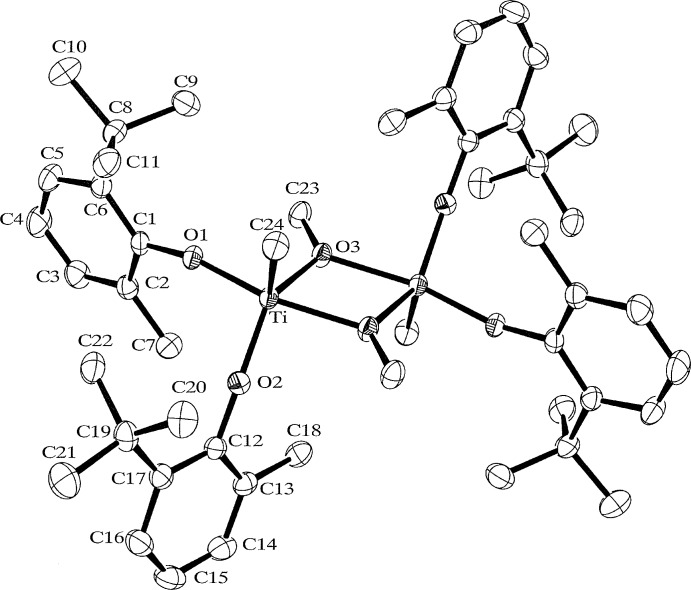
ORTEP diagram of molecule, at the 50% probability level, showing the numbering system.

## supplementary crystallographic information

### 1. Comment

Titanium complexes containing phenolato ligands (OAr) or alkoxo ligands (OR) as well as an alkyl ligand have been described (Janas *et al.* 2005; Janas *et al.* 2004; Zhang 2007*a*,*b*; Kobylka *et al.* 2007) but complexes containing both oxygen ligand sets and an alkyl ligand are not known. The chemistry of phenolato and alkylato complexes has been known for many years (Bradley *et al.* 1978) and in particular alkoxy bridged *bis-*phenolato dititanium complexes have been prepared (Ejfler *et al.* 2004). Several examples of X-ray crystal structures for titanium complexes containing terminal and bridging phenolato ligands have been reported (Gowda *et al.* 2009; Nielson *et al.* 2006; Svetich & Voge, 1972). During attempts to form *bis*-dimethyl *bis*-phenolato complexes of titanium for testing as olefin oligomerization and polymerization catalysts, in one case we reacted [TiCl_2_(OC_6_H_3_CMe_3_-2-Me-6)_2_] (Nielson *et al.* 2005; Santora *et al.* 1999) with two equivalents of methylmagnesium iodide and recrystallized the resulting product from petroleum spirit at low temperatures for a period of several months. A nice crystalline red coloured product was formed which was expected to be the *bis*-dimethyl complex [Ti(Me)_2_(OC_6_H_3_CMe_3_-2-Me-6)_2_]. However the X-ray crystal structural analysis showed it was the dimeric methoxy bridged complex [{Ti(Me)(µ-OMe)(OC_6_H_3_CMe_3_-2-Me-6)_2_}_2_] (1). Alkyl complexes of transition metal complexes are usually air sensitive and it was expected that the methoxo ligand had resulted from moist air entering the crystallization flask adventiciously and either water or oxygen inserting into a Ti–CH_3_ bond. A reaction where oxygen inserts into a Ti–CH_3_ bond has been reported (Zhang *et al.*, 2007*a*).

The structure of (1) is centrosymmetric and consists of an asymmetric, methoxy bridged dimer in which each Ti centre has two terminal phenolato ligands and a methyl ligand attached (Fig. 1). Each titanium atom has a distorted square pyramidal geometry in which the base of the square pyramid is made up by the oxygen atoms of the two *cis*-related terminal phenolato ligands and the oxygen atoms of the methoxy bridging system. A similar distorted square pyramidal structure is found in [{(tbop)Ti(Me)}_2_(µ-OMe)_2_] (tbop = 2,2-thiobis{4-(1,1,3,3- tetramethylbutyl)phenol} (Janas *et al.* 2005). The distortion in (1) is such that the O atom of one phenolato ligand and the *trans*-related oxygen of the methoxy bridge has a nearly linear disposition with the O(1)–Ti–O3^i^ bond angle at 162.64 (5)°. The other phenolato ligand oxygen and its *trans*-related methoxy ligand oxygen form a much more bent system where the O(2)–Ti–O(3) bond angle is 138.00 (5)°.

The two Ti atoms and the methoxide ligand oxygen atoms of the oxygen bridges (O3, O3^i^) are coplanar with O1 and O1^i^ lying on either side of the plane with displacements of 0.0234 (2) Å. The remaining oxygen of the square pyramidal base is displaced by -1.134 (2) Å.

The methyl group (C24) of the complex occupies the apical site of the square plane as similarly found in [{(tbop)Ti(Me}_2_(µ-OMe)_2_] (Janas *et al.* 2005). The distortion away from the square plane is also shown by the various C24–Ti– bond angles. The C24–Ti–O(3^i^) bond angle which involves the oxygen of the methoxy bridge is 90.33 (6)° whereas for the *trans*-related oxygen, O(1), which involves the phenolato ligand, the angle is 97.38 (7)° indicating that this oxygen pushes slightly closer to the methyl group than does the bridging oxygen. For the other bridging methoxy ligand the C(24)–Ti–O(3) bond angle is 112.91 (6) ° and the C(24)–Ti–O(2) bond angle involving the phenolato ligand oxygen is 104.51 (7) ° indicating that this oxygen pushes slightly closer to the methyl group than the bridging oxygen. The angles associated with the C(24)–Ti–O system thus show that for the methoxy bridge the C(24)–Ti–O(3) bond angle [112.91 (6) °] opens out considerably more than does the C(24)–Ti–O(3^i^) bond angle [90.33 (6) °]. The C(24)–Ti–O angles associated with the phenolato ligand are also very different with the C(24)–Ti–O(2) bond angle [104.51 (7) °] opened out more than the C(24)–Ti–O(1) bond angle [97.38 (7) °].

The methoxy bridging system has a Ti–O–Ti^i^ bond angle of 109.03 (5) °, an O(3)–Ti–O(3^i^) bond angle of 70.97 (5) ° with Ti–O(3)–C(23) and Ti–O(3)–C(23^i^) bond angles of 129.10 (10) and 121.77 (10) ° respectively which are similar to those found in other alkoxy bridged titanium dimers (Janas *et al.* 2005, 2004) The separation between the phenolato ligands is shown by the O(1)–Ti–O(2) angle of 102.02 (5) ° which is much wider than the O(3)–Ti–O(3^i^) angle associated with the methoxy bridge [70.97 (5) °] which means that the bridging system is compressed in comparison to the terminal phenolato ligands. This no doubt occurs since there is significant repulsion between the aromatic rings. However the two terminal phenolato ligand O atoms push away from the *cis*-related methoxo bridge O atoms to nearly equal extents [O(1)–Ti^i^–O(3) and O(2)–Ti^i^–O(3^i^) bond angles 91.68 (5) and 90.97 (5) ° respectively].

The aromatic rings of the *cis*-related phenolato ligands are rotated away from each other with the Ti–O(1)–C(1) and Ti–O(2)–C(12) bond angles being nearly equal [161.24 (10) and 160.66 (11)°]. In comparison, the related bridging *tris*-phenolato complex [{TiCl(OC_6_H_3_Me_2_-2,4)_2_(µ-OC_6_H_3_Me_2_-2,4)}_2_] has one terminal phenolato ligand Ti–O–C bond angle nearly linear [171.4 (1)°] and the other much more bent [Ti–O–C bond angle 138.8 (1)°] (Nielson *et al.* 2006). Although these angles are essentially the same in (1), one phenyl ring is rotated so that its face points inwards towards the other but slightly down and the other ring is rotated so that it points away from and slightly down from the former. The rotation is such that the *tert*-butyl substituents in the 2-position of the phenyl ring lie adjacent to each other, as do the two methyl substituents on the phenyl ring 6-position. For the two adjacent *tert*-butyl substituents the methyl carbons are related by a geared disposition which apparently allows a further gearing across the two substituents of the attached hydrogen atoms. Two methyl groups of both *tert*-butyl substituents also have a geared disposition with the adjacent carbon atom [C(24)] of the Ti methyl group.

The methoxy bridging system is asymmetric with the Ti–O(3) bond length [1.9794 (12) Å] significantly shorter than the Ti–O(3^i^) bond length [2.0603 (12) Å]. For the Ti–O bond lengths associated with the phenolato ligands the Ti–O1 and Ti–O2 bond lengths [1.8218 (11) and 1.8135 (11) Å respectively] differ only slightly from one another but are significantly shorter than the methoxy bridge system Ti–O bond lengths. These shorter bond lengths indicate that the phenolato ligands are the better π-donors to the metal and this is supported by the nearly linear Ti–O–C bond angles associated with them. In comparison the related bridging *tris*-phenolato complex [{TiCl(OC_6_H_3_Me_2_-2,4)_2_(µ-OC_6_H_3_Me_2_-2,4)}_2_] has one short Ti–O phenolato ligand bond length [1.757 (1) Å] which is related to the near linear Ti–O–C bond angle [171.4 (1)°] and thus is the stronger π-donor and one longer Ti–O phenolato ligand bond length [1.794 (2) Å] related to the more bent Ti–O–C bond angle [138.8 (1)°] and the poorer π-donor (Nielson *et al.*. 2006). The Ti–O–C bond angles associated with the asymmetric methoxy bridging system are much reduced in comparison [129.10 (10) and 121.77 (10)°] indicating the reduced π-donor ability in this coordination mode. Terminal alkoxo ligands have much shorter bond lengths and larger Ti–O–C bond angles and are usually stronger π-donor ligands than phenolato ligands which have the ability for competitive π-back-donation to the aromatic ring. However in all cases involving phenolato and alkoxo ligands there is a subtle interplay of the π-donor properties depending on coordination mode and geometry. The Ti–C bond length for the Ti—CH_3_ ligand [2.0878 (17) Å] is similar to the Ti–C bond length [2.078 (4) Å] found in [{(tbop)Ti(Me)}_2_(µ-OMe)_2_] (Janas *et al.* 2005).

### 2. Experimental

Methyl magnesium iodide (11.8 ml of a 1.076 mol/l solution, 12.7 mmole) in diethyl ether was added dropwise to a stirred suspension of [TiCl_2_(OC_6_H_3_CMe_3_-2-Me-6)_2_] (2.41 g, 5.79 mmole) in petroleum spirit (boiling point 40–60°) cooled in a dry-ice bath. The dry-ice bath was removed and the mixture stirred overnight. The solution was filtered, solvent removed and the residue extracted with hot petroleum spirit to give an orange-red solution. Reduction of the solvent volume and standing at -20° C gave a small quantity of the product as orange-red crystals. Found: C, 68.24; H, 9.10. C_48_H_72_O_6_Ti_2_ requires C, 68.56; H, 8.63. A crystal was chosen and the X-ray single crystal structure obtained.

### 3. Refinement

All H atoms were included in calculated positions and refined using a riding model [*U*(H)_eq_ = 1.2*U*C_eq_ for aromatic CH and *U*(H) = 1.5*U*(C) for methyl H atoms]. C—H distances of 0.96 Å and 0.93 Å were assumed for aromatic and methyl groups respectively.

### Crystal data

**Table:**

[Ti_2_(CH_3_)_2_(CH_3_O)_2_(C_11_H_15_O)_4_]	*F*(000) = 904
*M**_r_* = 840.80	V = 2386.4(8)
Monoclinic, *P*2_1_/*c*	*D*_x_ = 1.170 Mg m^−^^3^
Hall symbol: -P 2ybc	Mo *K*α radiation, λ = 0.71073 Å
*a* = 15.127 (3) Å	Cell parameters from 10380 reflections
*b* = 11.067 (2) Å	θ = 2–26°
*c* = 15.821 (3) Å	µ = 0.38 mm^−^^1^
β = 115.71 (3)°	*T* = 150 K
*V* = 2386.4 (8) Å^3^	Needle, yellow
*Z* = 2	0.48 × 0.20 × 0.18 mm

### Data collection

**Table:**

Siemens SMART diffractometer	4844 independent reflections
Radiation source: fine-focus sealed tube	4113 reflections with *I* > 2σ(*I*)
Graphite monochromator	*R*_int_ = 0.022
Area detector ω scans	θ_max_ = 26.4°, θ_min_ = 1.5°
Absorption correction: multi-scan (Blessing, 1995)	*h* = −9→18
*T*_min_ = 0.834, *T*_max_ = 0.953	*k* = −13→13
13277 measured reflections	*l* = −19→18

### Refinement

**Table:**

Refinement on *F*^2^	Secondary atom site location: difference Fourier map
Least-squares matrix: full	Hydrogen site location: inferred from neighbouring sites
*R*[*F*^2^ > 2σ(*F*^2^)] = 0.034	H-atom parameters constrained
*wR*(*F*^2^) = 0.089	*w* = 1/[σ^2^(*F*_o_^2^) + (0.0371*P*)^2^ + 1.0664*P*] where *P* = (*F*_o_^2^ + 2*F*_c_^2^)/3
*S* = 1.04	(Δ/σ)_max_ = 0.002
4844 reflections	Δρ_max_ = 0.30 e Å^−^^3^
263 parameters	Δρ_min_ = −0.30 e Å^−^^3^
0 restraints	Absolute structure: structure is centrosymmetric
Primary atom site location: structure-invariant direct methods	

### Special details

**Table:**

Geometry. All e.s.d.'s (except the e.s.d. in the dihedral angle between two l.s. planes) are estimated using the full covariance matrix. The cell e.s.d.'s are taken into account individually in the estimation of e.s.d.'s in distances, angles and torsion angles; correlations between e.s.d.'s in cell parameters are only used when they are defined by crystal symmetry. An approximate (isotropic) treatment of cell e.s.d.'s is used for estimating e.s.d.'s involving l.s. planes.
Refinement. Refinement of *F*^2^ against ALL reflections. The weighted *R*-factor *wR* and goodness of fit *S* are based on *F*^2^, conventional *R*-factors *R* are based on *F*, with *F* set to zero for negative *F*^2^. The threshold expression of *F*^2^ > σ(*F*^2^) is used only for calculating *R*-factors(gt) *etc*. and is not relevant to the choice of reflections for refinement. *R*-factors based on *F*^2^ are statistically about twice as large as those based on *F*, and *R*-factors based on ALL data will be even larger.

### Fractional atomic coordinates and isotropic or equivalent isotropic displacement parameters (Å^2^)

**Table:**

	*x*	*y*	*z*	*U*_iso_*/*U*_eq_	
Ti	0.113050 (19)	0.99514 (2)	1.010663 (18)	0.02326 (9)	
O1	0.20285 (8)	0.87453 (10)	1.06042 (7)	0.0265 (2)	
O2	0.13233 (8)	1.04040 (10)	0.90976 (7)	0.0273 (2)	
O3	0.01427 (8)	0.90487 (9)	1.03621 (8)	0.0275 (2)	
C1	0.25027 (11)	0.76640 (14)	1.07086 (11)	0.0266 (3)	
C2	0.21767 (12)	0.69000 (15)	0.99229 (12)	0.0306 (3)	
C3	0.26384 (14)	0.57900 (16)	0.99989 (14)	0.0404 (4)	
H3	0.2419	0.5270	0.9487	0.048*	
C4	0.34152 (16)	0.54566 (18)	1.08239 (15)	0.0488 (5)	
H4	0.3730	0.4722	1.0865	0.059*	
C5	0.37277 (14)	0.62199 (18)	1.15939 (14)	0.0437 (5)	
H5	0.4252	0.5979	1.2148	0.052*	
C6	0.32879 (12)	0.73322 (15)	1.15704 (12)	0.0312 (4)	
C7	0.13366 (13)	0.72806 (16)	0.90185 (12)	0.0356 (4)	
H7A	0.1204	0.6657	0.8557	0.053*	
H7B	0.1506	0.8014	0.8799	0.053*	
H7C	0.0764	0.7414	0.9122	0.053*	
C8	0.36418 (12)	0.81365 (17)	1.24508 (12)	0.0364 (4)	
C9	0.27979 (14)	0.8334 (2)	1.27289 (13)	0.0468 (5)	
H9A	0.2270	0.8749	1.2232	0.070*	
H9B	0.3027	0.8810	1.3291	0.070*	
H9C	0.2569	0.7566	1.2837	0.070*	
C10	0.44889 (16)	0.7549 (2)	1.32983 (14)	0.0556 (6)	
H10A	0.4281	0.6778	1.3425	0.083*	
H10B	0.4671	0.8061	1.3837	0.083*	
H10C	0.5042	0.7440	1.3160	0.083*	
C11	0.40205 (13)	0.93433 (18)	1.22650 (13)	0.0414 (4)	
H11A	0.4591	0.9204	1.2159	0.062*	
H11B	0.4189	0.9864	1.2798	0.062*	
H11C	0.3519	0.9719	1.1721	0.062*	
C12	0.13202 (12)	1.03778 (14)	0.82316 (11)	0.0272 (3)	
C13	0.04673 (13)	0.99423 (14)	0.74772 (11)	0.0307 (3)	
C14	0.04534 (15)	0.99010 (16)	0.65910 (12)	0.0381 (4)	
H14	−0.0108	0.9633	0.6081	0.046*	
C15	0.12567 (16)	1.02501 (17)	0.64586 (13)	0.0437 (5)	
H15	0.1243	1.0197	0.5866	0.052*	
C16	0.20860 (15)	1.06815 (17)	0.72079 (13)	0.0399 (4)	
H16	0.2620	1.0922	0.7104	0.048*	
C17	0.21512 (12)	1.07692 (15)	0.81156 (12)	0.0312 (4)	
C18	−0.04102 (13)	0.95336 (18)	0.76132 (13)	0.0381 (4)	
H18A	−0.0224	0.8880	0.8056	0.057*	
H18B	−0.0915	0.9266	0.7024	0.057*	
H18C	−0.0652	1.0194	0.7846	0.057*	
C19	0.30757 (13)	1.12730 (16)	0.89323 (13)	0.0365 (4)	
C20	0.28073 (14)	1.24147 (17)	0.93236 (14)	0.0442 (5)	
H20A	0.2569	1.3024	0.8844	0.066*	
H20B	0.3378	1.2710	0.9849	0.066*	
H20C	0.2307	1.2223	0.9522	0.066*	
C21	0.38714 (16)	1.1638 (2)	0.86143 (17)	0.0560 (6)	
H21A	0.4056	1.0945	0.8363	0.084*	
H21B	0.4436	1.1944	0.9142	0.084*	
H21C	0.3617	1.2253	0.8141	0.084*	
C22	0.35367 (14)	1.03064 (18)	0.97023 (14)	0.0423 (4)	
H22A	0.3073	1.0078	0.9938	0.063*	
H22B	0.4115	1.0629	1.0205	0.063*	
H22C	0.3709	0.9610	0.9444	0.063*	
C23	0.02098 (13)	0.78584 (15)	1.07531 (13)	0.0343 (4)	
H23A	−0.0171	0.7303	1.0264	0.051*	
H23B	−0.0038	0.7875	1.1218	0.051*	
H23C	0.0883	0.7603	1.1038	0.051*	
C24	0.18633 (13)	1.12601 (16)	1.11211 (12)	0.0358 (4)	
H24A	0.2486	1.1433	1.1123	0.054*	
H24B	0.1965	1.0965	1.1727	0.054*	
H24C	0.1476	1.1984	1.0980	0.054*	

### Atomic displacement parameters (Å^2^)

**Table:**

	*U*^11^	*U*^22^	*U*^33^	*U*^12^	*U*^13^	*U*^23^
Ti	0.02042 (14)	0.02367 (15)	0.02448 (14)	0.00311 (11)	0.00862 (11)	−0.00058 (11)
O1	0.0221 (5)	0.0268 (5)	0.0287 (6)	0.0042 (4)	0.0091 (4)	0.0002 (4)
O2	0.0261 (6)	0.0295 (6)	0.0265 (5)	0.0006 (5)	0.0117 (5)	−0.0002 (4)
O3	0.0250 (6)	0.0233 (5)	0.0343 (6)	0.0042 (4)	0.0130 (5)	0.0042 (5)
C1	0.0221 (7)	0.0264 (8)	0.0340 (8)	0.0035 (6)	0.0146 (7)	0.0029 (6)
C2	0.0318 (8)	0.0275 (8)	0.0361 (9)	0.0025 (7)	0.0181 (7)	0.0019 (7)
C3	0.0483 (11)	0.0311 (9)	0.0466 (10)	0.0047 (8)	0.0250 (9)	−0.0019 (8)
C4	0.0524 (12)	0.0351 (10)	0.0623 (13)	0.0200 (9)	0.0279 (11)	0.0086 (9)
C5	0.0361 (10)	0.0461 (11)	0.0458 (11)	0.0163 (8)	0.0147 (8)	0.0141 (9)
C6	0.0247 (8)	0.0353 (9)	0.0349 (9)	0.0040 (7)	0.0142 (7)	0.0066 (7)
C7	0.0389 (10)	0.0303 (8)	0.0335 (9)	−0.0005 (7)	0.0120 (8)	−0.0061 (7)
C8	0.0253 (8)	0.0495 (11)	0.0296 (8)	0.0038 (8)	0.0075 (7)	0.0053 (8)
C9	0.0407 (10)	0.0690 (14)	0.0337 (9)	0.0009 (10)	0.0189 (8)	−0.0020 (9)
C10	0.0438 (12)	0.0709 (15)	0.0378 (10)	0.0110 (11)	0.0044 (9)	0.0101 (10)
C11	0.0283 (9)	0.0491 (11)	0.0393 (10)	−0.0033 (8)	0.0076 (8)	−0.0040 (8)
C12	0.0317 (8)	0.0230 (7)	0.0278 (8)	0.0060 (6)	0.0137 (7)	0.0018 (6)
C13	0.0353 (9)	0.0247 (8)	0.0296 (8)	0.0047 (7)	0.0117 (7)	0.0012 (6)
C14	0.0492 (11)	0.0313 (9)	0.0288 (8)	0.0057 (8)	0.0121 (8)	−0.0025 (7)
C15	0.0651 (13)	0.0401 (10)	0.0325 (9)	0.0075 (9)	0.0273 (9)	0.0009 (8)
C16	0.0498 (11)	0.0369 (10)	0.0440 (10)	0.0055 (8)	0.0304 (9)	0.0063 (8)
C17	0.0353 (9)	0.0256 (8)	0.0360 (9)	0.0060 (7)	0.0185 (7)	0.0038 (7)
C18	0.0306 (9)	0.0422 (10)	0.0332 (9)	−0.0020 (8)	0.0059 (7)	−0.0002 (8)
C19	0.0291 (9)	0.0380 (9)	0.0453 (10)	−0.0012 (7)	0.0188 (8)	0.0004 (8)
C20	0.0377 (10)	0.0362 (10)	0.0555 (12)	−0.0072 (8)	0.0172 (9)	−0.0090 (9)
C21	0.0419 (11)	0.0667 (14)	0.0670 (14)	−0.0070 (10)	0.0308 (11)	0.0053 (11)
C22	0.0286 (9)	0.0492 (11)	0.0478 (11)	0.0050 (8)	0.0154 (8)	0.0035 (9)
C23	0.0310 (9)	0.0265 (8)	0.0450 (10)	0.0044 (7)	0.0160 (8)	0.0091 (7)
C24	0.0334 (9)	0.0330 (9)	0.0364 (9)	−0.0003 (7)	0.0107 (7)	−0.0080 (7)

### Geometric parameters (Å, º)

**Table:**

Ti—O1	1.8218 (11)	C7—H7A	0.9600
Ti—O2	1.8135 (11)	C7—H7B	0.9600
Ti—O3	1.9794 (12)	C7—H7C	0.9600
Ti—O3^i^	2.0603 (12)	C3—C4	1.375 (3)
Ti—C24	2.0878 (17)	C3—H3	0.9300
Ti—Ti^i^	3.2897 (9)	C5—C4	1.386 (3)
O3—Ti^i^	2.0603 (12)	C5—H5	0.9300
O1—C1	1.3678 (18)	C11—H11A	0.9600
O2—C12	1.3683 (18)	C11—H11B	0.9600
O3—C23	1.4401 (19)	C11—H11C	0.9600
C1—C2	1.404 (2)	C22—H22A	0.9600
C1—C6	1.414 (2)	C22—H22B	0.9600
C17—C16	1.400 (2)	C22—H22C	0.9600
C17—C12	1.415 (2)	C24—H24A	0.9600
C17—C19	1.539 (3)	C24—H24B	0.9600
C19—C20	1.537 (3)	C24—H24C	0.9600
C19—C22	1.542 (3)	C13—C14	1.394 (2)
C19—C21	1.546 (2)	C20—H20A	0.9600
C12—C13	1.409 (2)	C20—H20B	0.9600
C6—C5	1.392 (2)	C20—H20C	0.9600
C6—C8	1.540 (2)	C15—C14	1.375 (3)
C18—C13	1.504 (2)	C15—H15	0.9300
C18—H18A	0.9600	C4—H4	0.9300
C18—H18B	0.9600	C10—H10A	0.9600
C8—C11	1.531 (3)	C10—H10B	0.9600
C8—C9	1.536 (2)	C10—H10C	0.9600
C8—C10	1.540 (3)	C14—H14	0.9300
C23—H23A	0.9600	C9—H9A	0.9600
C23—H23B	0.9600	C9—H9B	0.9600
C23—H23C	0.9600	C9—H9C	0.9600
C2—C3	1.392 (2)	C21—H21A	0.9600
C2—C7	1.504 (2)	C21—H21B	0.9600
C16—C15	1.384 (3)	C21—H21C	0.9600
C16—H16	0.9300		
			
O1—Ti—O2	102.02 (5)	H7A—C7—H7B	109.5
O1—Ti—O3	91.68 (5)	C2—C7—H7C	109.5
O2—Ti—O3	138.00 (5)	H7A—C7—H7C	109.5
O1—Ti—O3^i^	162.64 (5)	H7B—C7—H7C	109.5
O2—Ti—O3^i^	90.97 (5)	C4—C3—C2	120.59 (18)
O3—Ti—O3^i^	70.97 (5)	C4—C3—H3	119.7
O1—Ti—C24	97.38 (7)	C2—C3—H3	119.7
O2—Ti—C24	104.51 (7)	C4—C5—C6	122.60 (17)
O3—Ti—C24	112.91 (6)	C4—C5—H5	118.7
O3^i^—Ti—C24	90.33 (6)	C6—C5—H5	118.7
O1—Ti—Ti^i^	127.98 (4)	C8—C11—H11A	109.5
O2—Ti—Ti^i^	117.24 (5)	C8—C11—H11B	109.5
O3—Ti—Ti^i^	36.30 (3)	H11A—C11—H11B	109.5
O3^i^—Ti—Ti^i^	34.67 (3)	C8—C11—H11C	109.5
C24—Ti—Ti^i^	103.76 (6)	H11A—C11—H11C	109.5
C23—O3—Ti	129.10 (10)	H11B—C11—H11C	109.5
C23—O3—Ti^i^	121.77 (10)	C19—C22—H22A	109.5
Ti—O3—Ti^i^	109.03 (5)	C19—C22—H22B	109.5
C1—O1—Ti	161.24 (10)	H22A—C22—H22B	109.5
C12—O2—Ti	160.66 (11)	C19—C22—H22C	109.5
O1—C1—C2	117.10 (14)	H22A—C22—H22C	109.5
O1—C1—C6	121.39 (14)	H22B—C22—H22C	109.5
C2—C1—C6	121.51 (15)	Ti—C24—H24A	109.5
C16—C17—C12	116.15 (16)	Ti—C24—H24B	109.5
C16—C17—C19	121.37 (16)	H24A—C24—H24B	109.5
C12—C17—C19	122.47 (15)	Ti—C24—H24C	109.5
C20—C19—C17	109.47 (14)	H24A—C24—H24C	109.5
C20—C19—C22	111.10 (16)	H24B—C24—H24C	109.5
C17—C19—C22	110.29 (15)	C14—C13—C12	118.17 (16)
C20—C19—C21	107.17 (16)	C14—C13—C18	120.34 (16)
C17—C19—C21	111.94 (16)	C12—C13—C18	121.49 (15)
C22—C19—C21	106.81 (16)	C19—C20—H20A	109.5
O2—C12—C13	117.37 (15)	C19—C20—H20B	109.5
O2—C12—C17	120.40 (15)	H20A—C20—H20B	109.5
C13—C12—C17	122.23 (15)	C19—C20—H20C	109.5
C5—C6—C1	116.56 (16)	H20A—C20—H20C	109.5
C5—C6—C8	120.82 (16)	H20B—C20—H20C	109.5
C1—C6—C8	122.61 (15)	C14—C15—C16	119.93 (17)
C13—C18—H18A	109.5	C14—C15—H15	120.0
C13—C18—H18B	109.5	C16—C15—H15	120.0
H18A—C18—H18B	109.5	C3—C4—C5	119.73 (17)
C13—C18—H18C	109.5	C3—C4—H4	120.1
H18A—C18—H18C	109.5	C5—C4—H4	120.1
H18B—C18—H18C	109.5	C8—C10—H10A	109.5
C11—C8—C9	110.96 (16)	C8—C10—H10B	109.5
C11—C8—C6	110.04 (14)	H10A—C10—H10B	109.5
C9—C8—C6	109.59 (15)	C8—C10—H10C	109.5
C11—C8—C10	107.24 (16)	H10A—C10—H10C	109.5
C9—C8—C10	106.96 (16)	H10B—C10—H10C	109.5
C6—C8—C10	112.00 (16)	C15—C14—C13	121.02 (18)
O3—C23—H23A	109.5	C15—C14—H14	119.5
O3—C23—H23B	109.5	C13—C14—H14	119.5
H23A—C23—H23B	109.5	C8—C9—H9A	109.5
O3—C23—H23C	109.5	C8—C9—H9B	109.5
H23A—C23—H23C	109.5	H9A—C9—H9B	109.5
H23B—C23—H23C	109.5	C8—C9—H9C	109.5
C3—C2—C1	118.98 (16)	H9A—C9—H9C	109.5
C3—C2—C7	120.96 (16)	H9B—C9—H9C	109.5
C1—C2—C7	120.06 (14)	C19—C21—H21A	109.5
C15—C16—C17	122.47 (18)	C19—C21—H21B	109.5
C15—C16—H16	118.8	H21A—C21—H21B	109.5
C17—C16—H16	118.8	C19—C21—H21C	109.5
C2—C7—H7A	109.5	H21A—C21—H21C	109.5
C2—C7—H7B	109.5	H21B—C21—H21C	109.5

Symmetry code: (i) −*x*, −*y*+2, −*z*+2.
